# Deep Vein Thrombosis, Raynaud's Phenomenon, and Prinzmetal Angina in a Patient with Glanzmann Thrombasthenia

**DOI:** 10.1155/2012/156290

**Published:** 2012-12-31

**Authors:** Alan Nurden, Patrick Mercié, Pascal Zely, Paquita Nurden

**Affiliations:** ^1^Plateforme Technologique et d'Innovation Biomédicale, Hôpital Xavier Arnozan, 33600 Pessac, France; ^2^Service de Médecine Interne, Hôpital Saint André, 33000 Bordeaux, France; ^3^Clinique Saint Augustin, 33000 Bordeaux, France; ^4^CHU Timone, 13005 Marseille, France

## Abstract

Patients with Glanzmann thrombasthenia fail to form large platelet thrombi due to mutations that affect the biosynthesis and/or function of the **α**IIb**β**3 integrin. The result is a moderate to severe bleeding syndrome. We now report unusual vascular behaviour in a 55-year-old woman with classic type I disease (with no platelet **α**IIb**β**3 expression) and a homozygous *ITGA2B* missense mutation (E324K) affecting the terminal **β**-propeller domain of **α**IIb. While exhibiting classic bleeding symptoms as a child, in later life this woman first developed deep vein thrombosis after a long air flight then showed vascular problems characteristic of Raynaud's phenomenon, and finally this year she presented with chest pains suggestive of coronary heart disease. Yet while coronary angiography first showed a stenosis, this was not seen on a second examination when she was diagnosed with coronary spastic angina and Prinzmetal phenomenon. It is significant that the absence of platelet aggregation with physiologic agonists had not prevented any of the above cardiovascular or vascular diseases.

## 1. Introduction

Glanzmann thrombasthenia (GT) is a rare bleeding disorder characterized by a lack of platelet aggregation due to platelets with qualitative or quantitative deficiencies of the *α*IIb*β*3 integrin [1, 2]. Spontaneous mucocutaneous bleeding is the primary symptom but its severity varies considerably between patients and ranges from mild to life threatening. As patient care improves, more patients are becoming elderly and so the incidence of cardiac defects and thrombosis is of interest in a disease where macroscopic platelet aggregation fails to occur. We now describe a GT patient with an unique combination of vascular and thrombotic defects that extend our knowledge of the phenotypic heterogeneity that can accompany what is the principle inherited disorder of an integrin. They also imply that large platelet aggregates are not implicated in Raynaud's phenomenon and Prinzmetal angina.

## 2. Case Report

The propositus is a 56-year-old Algerian woman with classic type I GT. Previous studies by us have shown that her platelets lack detectable *α*IIb*β*3 due to a homozygous Glu324Lys (E324K) mutation in the *ITGA2B* gene [3]. As this mutation occurs in nonrelated families spread over 3 continents, the codon for E324 may be a mutational hotspot for the disease [3–5]. In vitro expression of K324*α*IIb with wild-type *β*3 in heterologous cells showed that the mutation permits pro-*α*IIb*β*3 complex formation but severely interferes with the trafficking and maturation of the integrin thereby preventing its transport to the cell surface [3]. Substitution of the negatively charged E with a positively charged K at position 324 modifies the *α*IIb *β*-propeller configuration essential for the processing of the complex. In fact, E324 forms a hydrogen bond with S291 of *β*3, the K324 substitution results in the loss of this bond and provides additional steric repulsive forces (illustrated in Figure 2 of [2]).

The patient shows all the typical signs of GT with no platelet aggregation in response to physiologic agonists and an absence of clot retraction. She is patient 9 in the clinical review of 64 patients by George et al. [1]. She has a normal platelet count and a typical prolonged bleeding time. The *β*3 subunit has no mutations and associates with *α*v to form *α*v*β*3, an important receptor in endothelial and other cell types [6]. *α*v*β*3 was normally detected in small amounts in platelets from the propositus confirming that her mutation does not prevent *α*v*β*3 biosynthesis [7]. Consanguinity is not documented but is suspected as both parents were from the same village. GT was diagnosed at an early age; her symptoms were typical of the disease and she was treated accordingly [1, 8]. In particular, she suffered as a young girl from repeated epistaxis requiring hospitalization and blood transfusions yet antiplatelet antibodies have never been found. Severe menstrual bleeding was controlled by birth control pills. She had a normal pregnancy in 1980 with vaginal delivery after platelet transfusions. After discontinuing birth control pills in view of trying for a second pregnancy menorrhagia was again a problem. Minor surgery for uterine synechia was performed without incident. Sheehan's syndrome (with decreased functioning of the pituitary gland) was diagnosed in 1984 with hypothyroidism and other hormonal problems that require medication. Overall, bleeding decreased as the propositus became older.

- *Deep vein thrombosis (DVT).* In 2005 our patient developed superficial DVT in a lower limb after a flight of 5 hours and a small blood clot was detected that disappeared without anticoagulation. DVT has been reported in several patients with GT [1, 9–12] and some of these cases are compared to our patient in Table 1; in two cases the DVT was associated with the presence of thrombotic risk factors such as Factor V Leiden that were not, however, present for our patient.
- *Raynaud's phenomenon and Prinzmetal angina.* In 2010, the propositus complained of discomfiture of the fingers and toes with discoloration. This was diagnosed as Raynaud's phenomenon, a condition caused by an exaggeration of vasomotor responses to cold or emotional stress. Hyperactivation of the sympathetic system causes extreme vasoconstriction and tissue hypoxia. The patient also complained of pain within knee joints and hips. There were no signs of an autoimmune response; tests for anti-HLA and anti-platelet antibodies were negative. Recently, the patient experienced severe chest pains and was hospitalized with a suspected acute coronary syndrome. This was surprising for a patient with GT where platelet thrombus formation does not occur. Although previous studies suggest that atherosclerosis can occur in this disease [13], only rare isolated reports refer to cardiac or ischemic problems in GT patients [14–16]. The initial coronary angiography indeed suggested a coronary artery stenosis of the left circumflex artery with moderate elevation of the troponin level. An initial treatment with beta-blockers was given but proved inefficient. However, on a later examination the stenosis was no longer seen and further testing showed that the patient was suffering from temporary spasms of the coronary artery typical of Prinzmetal angina [17, 18]. Her spastic coronaropathy was successfully treated with calcium blockers (Amlodipine and Verapamil).

## 3. Discussion

This patient adds to those who have experienced DVT thereby confirming that GT patients are not protected against venous thrombosis. As shown in Table 1, of those GT patients who have experienced DVT and for whom detailed reports are available, two have had recurrent events and while some of the patients have not been genotyped there is enough evidence to conclude that DVT can affect classic or variant forms, is independent of age or sex and can occur in patients with mutations of either the *ITGA2B* or *ITGB3* genes. It appears that DVT is a largely unrecognized risk in GT and, in this respect, GT may differ from other inherited platelet disorders [19]. Perhaps it is significant that a role for GPIb and VWF has been proposed in venous thrombosis while the ability of GT platelets to bind to fibrin is well known [20, 21].

The coassociation of Prinzmetal angina with Raynaud's syndrome may suggest an underlying cause for these disorders for which blood vessel spasm is an underlying feature [17]. This must occur independently of the *ITGA2B* mutation causing GT in our patient, as this gene is not expressed in endothelial cells. While a role for activated platelets cannot be excluded, the total absence of platelet aggregation in our patient means that *α*IIb*β*3-dependent thrombus formation cannot be a cause of either phenomena. Meanwhile, a survey of the literature suggests that the association of pathologies described for this patient is unique for GT thereby extending the phenotypic heterogeneity of the disease. Prinzmetal angina should be excluded in GT patients presenting with sporadic chest pains suggestive of a coronary artery syndrome.

## Figures and Tables

**Table 1:** Patients with DVT described in the literature and this study.

Patient	Bleeding	Platelet Aggreg	Residual *α*IIb*β*3	Mutation	Ref	Comments
N° 1—elderly male	Mild	Absent	50%	*β*3-Ser752Pro	[12]	Severe proximal DVT and pulmonary embolism after long airflight—treated with LMWH
N° 2—adult male	GI bleeding	Absent	Absent/low	Not known	[9]	Recurrent (3X) proximal DVT in same leg. Factor V Leiden. Treated with LMWH
N° 3—adult male	Mild	Absent	Absent	Not known	[10]	Recurrent DVT & possible pulmonary embolism. Factor V Leiden. Treated with heparin and warfarin
N° 4—2-yr-old girl	Repeated epistaxis	Absent	Much reduced	Not known	[11]	Proximal DVT after platelet transfusions (femoral catheter) and rFVIIa. No anticoagulation
N° 5—adult woman	Moderate	Absent	Trace amounts	*β*3-Cys457Tyr	[1] (patient 11) [22]	Single episode treated with heparin
N° 6—adult woman	Severe when child	Absent	Absent	*α*IIb-Glu324Lys	[1] (patient 9) and this study	Single episode without treatment Raynaud's phenomenon and Prinzmetal angina

Patients were included on the basis of a detailed literature report and of the availability of clinical and biological data. Mutations are given when known. LMWH: low molecular weight heparin. rFVIIa: recombinant factor VIIa. Aggreg: aggregation.
